# Editorial In this issue

**DOI:** 10.1590/S1677-5538.IBJU.2015.06.01

**Published:** 2015

**Affiliations:** Divisão de Urologia do A.C. Camargo Cancer Center Fundação A. Prudente, São Paulo, Brasil

This is the last 2015 issue of the 41th year of publication of the International Brazilian Journal of Urology. During this year, we have published more than 200 articles, and the number of submissions has increased continuously, requiring an intensive work of our reviewers.

In this number, we publish a controversy in the difference of opinion section (pages. 1043-1045): Experts (Dr. Vieira from Brazil and Drs. Chilles and Schlegel from US) discuss the pros and cons of vasectomy reversion as the first step in a vasectomized man who wants to father again in a new marriage.

In page 1049 a review article confirms the effectiveness of the use of alpha-1 blocker drugs in the medical expulsive therapy for ureteral calculus in children. The drugs increased the probability of calculus expulsion, independent of its size (cut –off 5mm), however it was included only three satisfactory studies in this review, showing the need for more investigation in this area.

There are few studies regarding adjuvant chemotherapy for locally advanced upper tract urothelial carcinoma. The group from Seoul, Korea has shown no benefits with cisplatin based therapy after nephroureterectomy in a retrospective cohort of 72 patients (page 1067).

Recently, it was demonstrated that the cessation of preventive aspirin use is not necessary in the majority of surgeries, except in prostatic and brain procedures. The group from New Delhi, India, concluded in almost 700 patients that aspirin cessation is not necessary during the realization of trans-rectal prostatic biopsies (12 cores, page 1096).

Married status seems to be a protective factor for oncologic patients, as already demonstrated for prostate cancer, penile cancer and other prevalent neoplasms. The groups from Georgia University and Roswell Park (US) concluded that married patients presented better survival rates also in adrenocortical carcinoma. Single, widowed or divorced patients presented a 25-30% higher risk of mortality in this usually aggressive tumor (page 1108).

In page 1126, the Washington University group demonstrated that hemorrhagic cystitis in stem cell transplant patients presents higher mortality when continuous bladder irrigation is required.

In neuro-urology, an Egyptian group compared the results of intra vesical injection of 100 versus 200 units of botulin toxin A in the treatment of neurogenic bladder; and there is an experimental study from Toulouse and London researchers that investigated for the first time in humans the immunohistochemical expression of SPK-1 (a bladder wall modulator) in the vesical wall of patients with neurogenic bladder dysfunctions (page 1141).

Villar's group from Federal University of Pernambuco reported satisfactory tissular interaction of a new material, the cellulose exopolysaccharide (CEC) inserted as pubovaginal sling in rats.

To reinforce the debate regarding open versus laparoscopic nephrectomy, a group from Ankara (page 1202) has shown that pulmonary function was less affected in patients who underwent laparoscopic rather than open surgeries.

Video section presents a technique associating resection of the urethral plate and an elongated ventral buccal graft for patients with extensive obliterans urethral stenosis, resulting in 81% of success in 36 Serbian patients.

The group from Cleveland Clinic investigated prostatic biopsies in patients under testosterone reposition. Additionally, we have robotic articles from US and Italy, prognostic evaluation of localized prostatic cancer patients from Sao Paulo University, an Iranian investigation of interleukin 8 as a serum marker for children with febrile urinary tract infections, Chinese experimental studies etc…

We wish a happy and productive new year for all worldwide Int Braz J Urol contributors, friends and relatives.

